# Understanding the Influence of Initial Values of College Students in Shaping Pro-Environmental Behavioral Intention

**DOI:** 10.3390/ijerph19159730

**Published:** 2022-08-07

**Authors:** Yanqing Song, Han Bao, Shan Shen

**Affiliations:** School of Public Policy and Administration, Chongqing University, Chongqing 400044, China

**Keywords:** pro-environment, behavioral intention, social values, personal values, college students, TAM

## Abstract

Pro-environmental behaviors are rooted in values, and understanding the initial values among college students is pivotal in developing educational strategies to improve their pro-environmental behavior. However, most pro-environmental behavior studies fail to consider the social values and personal values as different dimensional or even conflicting values. This study integrated two distinct values, namely perceived social values and perceived personal values, with the technology acceptance model (TAM) to examine how different values shape college students’ pro-environmental behavioral intentions. The proposed model was then empirically validated using survey data from 245 responses from freshmen students at a University in Chongqing. The findings reveal that while perceived social values and perceived personal values are both positively related to behavioral intention, the effect sizes of the former are much larger. Our findings highlight that higher institutions and instructors should continue shaping the prosocial values among college students and create personal values from pro-environmental behavior to reduce the detrimental impact on the environment and achieve sustainability.

## 1. Introduction

Promoting pro-environmental behavior is important to reduce our detrimental impact on the environment and achieve sustainability. This is because many environmental changes are rooted in human actions [1]. More pro-environmental activities are prone to contribute to positive environmental changes, while lacking them may otherwise lead to negative changes [2,3,4,5]. In this regard, pro-environmental behavior should be encouraged among the massive public to protect the environment from further deterioration, and particularly among the young generation who is enduring the burden of the past and current environmental problems and will need to face and cope with future environmental changes [4]. Nevertheless, Grønhøj and Thøgersen [6] argued that, despite the fact that they are more competent, the young generation may be reluctant to conduct pro-environment behavior because they have not yet formed a strong intention in pro-environmental behavior. Further, Karp [7] argued that college is an important period for value shaping, and compared to the general public, such values play a more critical role in shaping college students’ pro-environmental behavior intentions. Thus, understanding how values shape pro-environmental behavior intentions and developing appropriate coping strategies to cultivate their pro-environmental intentions is extremely important for the young generation (e.g., college students) (Levine and Strube, 2012; Whitley et al., 2018) [8,9].

Extensively studies have attempted to reveal the pro-environmental behavioral intentions in fields such as recycling (Chan and Bishop, 2013; Wan et al., 2017) [2,3], waste management (Ma et al., 2017; Wu et al., 2017) [10,11], green energy consumption, low-carbon lifestyle (Büchs et al., 2015) [5], etc. These studies have often used different theories as the lens to understand the motivational antecedents of pro-environmental behavior, such as the theory of planned behavior (TPB) (Ajzen, 1991) [12], Norm Activation Model (NAM) (Schwartz, 1977) [13], values-belief-norm theory (VBNT) of environmentalism (Stern et al., 1999; Whitley et al., 2018) [8,14], and protection motivation theory (PMT) (Rogers, 1975) [15]. These studies provide solid theoretical bases for revealing the motivational factors for behavioral intention; however, they are majorly composed of the general public (Alzubaidi et al., 2021; Bamberg and Möser, 2007; Barbaro and Pickett, 2016; Czajkowski et al., 2017; Li et al., 2019) [16,17,18,19,20]. Only sporadic studies (Boyce and Geller, 2001; De Leeuw et al., 2015; Effendi et al., 2020; Shafiei and Maleksaeidi, 2020) [1,4,21,22] have been targeted at a young group. Yet, individuals’ values and how they motivate pro-environmental behavioral intentions are still unclear.

Despite the classic taxonomy of values by Bardi and Schwartz (2003) [23], we argued that for the young community, a significant conflict lies between social values and personal values (Belschak and Den Hartog, 2010; Hilbig et al., 2014) [24,25].Particularly in pro-environmental activity, prosocial values (Kaiser and Byrka, 2011) [26] is the key motivation of altruistic behavior such as pro-environmental behavior, and understanding how prosocial values motivate pro-environmental behavior intentions can be leveraged to promote engagement among the young group (e.g., college students) through proper educational intervention. As college is one of the critical periods for value shaping, understanding their motivation factors for pro-environmental behaviors can better reveal their pro-social values, which can in turn mirror the educational outcomes and further point to the needs in value shaping. In this regard, we argued that separately investigating the influence of social values and personal values can provide nuanced insights into how values motivate students’ pro-environmental behavioral intention and are deemed a starting point for developing better education strategies for enhanced pro-environmental behaviors. Thus, this study is conducted to address the following research question: How do initial social values and personal values influence pro-environmental behavior intention among college freshmen students?

To this end, we chose to use TAM as the theoretical lens to provide nuanced insights into students’ cognitive process in behavior intention in pro-environmental activity. Upon the TAM constructs, we substitute perceived usefulness as the perceived values to probe into the correlation between the values on college students’ pro-environmental behavioral intention. Specifically, we further adopted social values and personal values as two motivational factors of the model to reveal how the two conflicting values influence behavioral intention. In this study, we specially targeted the freshmen students rather than the general population of college students because we were interested in examining the initial values of the students before any education intervention from higher education. Thus, we controlled for the education intervention and recruited 245 freshmen college students from a university in China as the respondents. The findings reveal that perceived social values is the key motivational factor for pro-environmental behavior; the influence of perceived personal values is small but non-negligible. Our findings suggest that higher institutions should continue shaping the prosocial values among college students and create personal values from pro-environmental behavior to reduce the detrimental impact on the environment and achieve sustainability.

The remainder of this paper is structured as follows. The theoretical background is presented in Section 2. Section 3 and Section 4 describe the research hypotheses and method, respectively. This is followed by analysis of the research results and discussion of the practical and theoretical implications in Section 5. The conclusion, limitation, and scope for future research are summarized in the final section.

## 2. Theoretical Background

### 2.1. Pro-Environmental Behavior and Antecedents of Pro-Environmental Behavior

Pro-environmental behavior is widely referred to as the intentional action to impose positive impacts or reduce the negative impacts on the environment (Stern, 2000) [27]. Pro-environmental behaviors can include recycling (Chan and Bishop, 2013; Wan et al., 2017) [2,3], waste management (Ma et al., 2017; Wu et al., 2017) [10,11], green energy consumption, low carbon lifestyle (Büchs et al., 2015) [5], etc. In all, pro-environmental behavior is rooted in human actions (Vlek and Steg, 2007) [28]; thus, in psychology, extensive studies have attempted to probe into the cognitive process of pro-environmental behavior through the lens of psychological theory such as the theory of planned behavior (TPB) (Ajzen, 1991) [12], Norm Activation Model (NAM) (Schwartz, 1977) [13], values-belief-norm theory (VBNT) of environmentalism (Stern et al., 1999) [14], and protection motivation theory (PMT) (Rogers, 1975) [15].

### 2.2. Theory of Planned Behavior

TPB is a well-established theoretical framework for understanding pro-environmental behavior (Alzubaidi et al., 2021) [16]. It hypothesizes that individuals’ pro-environmental intention is determined by three constructs, namely attitudes, perceived behavior control, and subjective norms. In other words, TPB emphasizes that individuals’ competence (perceived behavior control), social influence (subjective norm), and individual attitudes are three antecedents of pro-environmental behavior. TPB is extensively used as a theoretical foundation of pro-environmental behavior (De Leeuw et al., 2015; Hameed et al., 2019; Mancha and Yoder, 2015; Shin et al., 2018) [4,29,30,31]; however, there are two main critiques for utilizing TPB for revealing the pro-environmental behavior. First, the attitude is too general to explain individuals’ motivation for pro-environmental behavior. Individuals’ attitudes toward pro-environmental behavior could be further driven by factors such as environmental concerns (Fransson and Gärling, 1999) [32], which remain unaddressed in TPB. Further, TPB does not incorporate intention into the model, which is argued to be important in understanding an individual’s pro-environmental behavior (Bamberg and Möser, 2007; Onwezen et al., 2013) [17,33].

### 2.3. Norm Activation Model

Another vein of scholars explored the pro-environmental behavior based on the NAM. NAM emphasizes that pro-environmental behavior is determined by personal norms, which is further formulated by the awareness that performing (or not performing) the particular behavior has certain consequences, and the feeling of responsibility for performing the specific behavior (Schwartz, 1977) [13]. Despite its wide applications (Shin et al., 2018; Zhang et al., 2013) [31,34], NAM is criticized for simplifying the motivation of personal norms as anticipated guilt, and fails to provide more nuanced insights into other motivation content of pro-environmental behavior (Thøgersen, 2006) [35].

### 2.4. Values-Belief-Norm Theory

Further, VBNT posits that values and the moral norm are the central motivation for pro-environmental behavior (Stern et al., 1999) [14]. Specifically, it assumes values, beliefs, norms, and behaviors in a causal chain that finally influence pro-environmental behavior (López-Mosquera and Sánchez, 2012) [36]. As such, individuals engage in pro-environmental behavior because their feeling of responsibility nudges their belief in taking action and further nudges their feeling of moral obligation to pro-environmental behavior. Nevertheless, despite the explanatory power of VBNT in formulating the psychological process of pro-environmental behavior (Estrada et al., 2017; Izagirre-Olaizola et al., 2015) [37,38], it is criticized for not including external factors such as social norms or the convenience of the process (Awais et al., 2022) [39].

### 2.5. Protection Motivation Theory

Another popular theory for explaining pro-environmental behavior is the PMT (Rogers, 1975) [15]. Compared to the previous theoretical framework, Shafiei and Maleksaeidi (2020) [1] argued that PMT provides more insights into the motivation of pro-environmental behavior. Specifically, PMT considered pro-environmental behavior as a cost analysis result of two cognitive processes, namely threat appraisal and coping appraisal (Rogers, 1975) [15]. PMT is extensively deployed to explain the adaptive behavior (e.g., protection) after changes (e.g., threats) (Bockarjova and Steg, 2014; Keshavarz and Karami, 2016) [40,41]; however, Kothe et al. (2019) [42] argued the causality between PMT constructs is questioned because a change in the PMT construct was of insufficient magnitude to lead to a detectable change in intention.

Most of the existing framework on pro-environmental behavior is targeted at the general public (Alzubaidi et al., 2021; Bamberg and Möser, 2007; Barbaro and Pickett, 2016; Czajkowski et al., 2017; Li et al., 2019) [16,17,18,19,20], and antecedents of pro-environmental can be classified as an external variable (e.g., social norms, threats) and internal variable (e.g., efficacy, rewards, values, belief) (Li et al., 2019) [19]. By comparison, researchers have implied that young people are less committed to pro-environmental behavior, despite often holding more favorable environmental attitudes and higher competence (De Leeuw et al., 2015; Grønhøj and Thøgersen, 2012) [4,5,6]. Thus, studies revolving around the general public may fail to provide nuanced insights into the motivation of college students’ pro-environmental behavior.

## 3. Hypotheses Development

Only a few studies attempted to investigate the pro-environmental behavior of the young generation (e.g., high-school students and university students) (Boyce and Geller, 2001; De Leeuw et al., 2015; Shafiei and Maleksaeidi, 2020) [1,4,21]. Specifically, De Leeuw et al. (2015) [4] posited that, among all antecedents, the subjective values that young people hold towards the environment could have a prominent role in fostering their pro-environmental behavior. In light of this, it is important to investigate how these values affect their pro-environmental behavior. In this study, we integrated the technology acceptance model (TAM) with values to explore college students’ motivation for pro-environmental behavior.

### 3.1. Technology Acceptance Model

TAM was proposed by Davis (1989) [43] to explain users’ behavioral intentions. Derived from the Theory of Reasoned Action (TRA) and the Theory of Planned Behavior (TPB), TAM presumes that users’ behavior is determined by their behavior intention, which is further influenced by an external stimulus (e.g., system feature and capabilities) (Davis, 1985) [44]. The external stimulus can be further formulated using two constructs, where perceived usefulness refers to “a user believes in the existence of a positive use-performance” while the perceived ease of use refers to a user who believes in the effortlessness of the system use (Davis, 1985, 1989) [43,44]. Upon this classic formulation, TAM is extended for the purpose of explaining the behavior intention in numerous fields such as business systems (Lee, 2009) [45], learning systems (Hu et al., 2022) [46], and automated vehicles (Zhang et al., 2019) [47]. Particularly for the pro-environmental behavior, existing studies based on the TAM framework are majorly resolving Green IT (Mishra et al., 2014; Yoon, 2018) [48,49]. Nevertheless, in both studies, the influence of the values is examined through other constructs such as perceived usefulness, environmental beliefs, or personal beliefs, which fail to provide a direct connection between values and behavior.

### 3.2. Perceived Social Values, Perceived Personal Values, and Behavioral Intention

In this study, we aimed to probe into the direct influence of values on pro-environmental behavior in order to provide more nuanced insights into a better educational intervention for shaping students’ values on pro-environmental behavior. Values have long been considered an important antecedent of behaviors, and research has demonstrated that values are a motivational construct of behavior (Bardi and Schwartz, 2003; Rokeach, 1973) [23,50]. We argued that people are likely to act in accordance with their values. In doing so, we have revised the constructs in the TAM model to fit in the pro-environmental context. Specifically, perceived usefulness refers to the perception that using the new technology will improve performance (Davis et al., 1989) [51]. In this study, it is expected to explore how the beliefs are forming; thus, we substituted the construct of perceived usefulness with perceived values. Further, in deriving how different values (e.g., social values, personal values) influence the behavior, the perceived values are further separated into two constructs, namely perceived social values and perceived personal values. Numerous studies have indicated that both social values and personal values are related to pro-environmental behavioral intention (Bogaert et al., 2008; Hilbig et al., 2014) [24,52] and students are likely to behave in accordance with their values (Belschak and Den Hartog, 2010) [25]. We conjectured accordingly that:

**Hypothesize 1a (H1a).**  *College students’ perceived personal values are positively related to their pro-environmental behavioral intention.*

**Hypothesize 1b (H1b).**  *College students’ perceived social values are positively related to their pro-environmental behavioral intention.*

### 3.3. Perceived Ease to Participate and Behavioral Intention

Further, in TAM, according to Davis, perceived ease of use is defined as “the degree to which a person believes that using a particular system would be free of effort” (Davis et al., 1989) [51]. Similar to the pro-environmental activity, we adapted the concept of perceived ease of use as perceived ease to participate, referring to the degree to which college students believe that participating in pro-environmental behavior is free of effort. Extensive studies have implied that the perceived ease of participation is related to behavior intention (Lee et al., 2017; Videras et al., 2012) [53,54]. For instance, Kaiser and Byrka (2011) [26] posited that the smallest inconvenience can be enough to stop a person from engaging in a behavior. Therefore, we argued that if pro-environmental behavior is free of effort, it will encourage college students’ intention to participate, while if it is not, it will impede their willingness to participate. Moreover, existing studies on pro-environmental behavior (e.g., Green IT adoption) (Yoon, 2018) [48] have indicated that perceived ease of use has a direct effect on perceived usefulness. Similarly, we assumed that the direct effect can be extended for our research model such that perceived ease to participate would have a direct effect on the perceived social values. We conjectured accordingly that:

**Hypothesize 2 (H2).**  *College students’ perceived ease of participation is positively related to their pro-environmental behavioral intention.*

**Hypothesize 3 (H3).**  *College students’ perceived ease of participation is positively related to perceived social values in pro-environmental behavior.*

## 4. Methodology

### 4.1. Participants and Procedure

This study follows a deductive approach, and hence the survey method is considered appropriate. We recruited freshman students that had experiences in pro-environment behaviors in a university in Chongqing, China. Specifically, before the survey began, we first presented a list of pro-environmental behaviors including waste classification, recycling, and energy conservation at home or at school. The students were asked whether they were aware that the above activities are good for the sustainability of the environment and if they had participated in any of these activities. If both questions were confirmed, we then invited them for the survey. The link to the survey was sent through WeChat, a popular instant message app, to college students. The survey started with a brief description of our aim and the clarification of the confidential issues. We clarified that the focus of this study was to better understand freshmen college students’ pro-environmental behavior and the survey was confidential, for research purposes only. To ensure consistency between the Chinese and the English version, we followed the back-translation method by Bhalla and Lin (1987) [55]. The wording, legibility, and suitability of the questionnaire were also checked by 3 graduate students and 2 undergraduate students before online delivery. Before distributing the questionnaire, a pilot study was conducted to test the reliability and validity of the survey items based on the results of a group of 20 offline-completed questionnaires from college students in China.

### 4.2. Construct Measurement

We adapted the measurement items from existing studies to suit the purpose of this study. The detailed constructs and measurements are listed in Table 1. All constructs were measured using a five-point Likert scale, anchored at 1 = strongly disagree and 5 = strongly agree.

### 4.3. Data Analysis

The study follows the two-step Structural Equational Model (SEM) approach recommended by Anderson and Gerbing (1988) [57] for the data analysis. The measurement items were first tested using the Confirmatory factor analysis to verify their reliability and validity. The relationships between constructs were then tested using SEM. (Hair, 2009) [58]. With comprehensive techniques of SEM, the AMOS 26 was adopted for the analysis.

## 5. Results

### 5.1. Descriptive Statics

This study was drawn from a survey of freshmen college students at a university in Chongqing, China. A total of 311 questionnaires were distributed. To ensure the validity of the survey, the incomplete or invalid (responses to questions have demonstrated certain patterns, such as identical) questionnaires were removed. We also set a response time threshold (2 min for the minimum and 15 min for the maximum) and removed those questionnaires that were filled outside the response time threshold. Finally, the survey yielded 245 valid responses (response rate of 79%) for further analysis. Descriptive statistics for the sample are represented in Table 2.

Given that behavior is rooted in an individual’s characteristics, we adopted a linear regression approach to identify the impacts of four demographics (independent variables) on the behavior intention (dependent variable). First, as previous studies imply that female students (Parrado et al., 2013) [59] and students who live in the rural area (Huddart-Kennedy et al., 2009) [60] are more favorable to pro-environment behavior, we added gender (women = 1) and home location (Urban = 1) as two independent variables. We posited that science or engineering students are more open to pro-environment activities and may have a higher intention, while business and management students are efficiency-oriented and may be less concerned about environmental issues. Thus, we further added academic disciplines variable (Science or Engineering = 1). Further, though all the respondents were freshmen students, we did consider the age difference; thus, age (age 18 = 1) was added to the regression model.

Our analytics results suggest the impacts of home location (β=−0.102, p>0.05), academic disciplines (β=0.017, p>0.05), and age (β=0.047, p>0.05) on pro-environmental behavioral intention were not significant. The gender difference (β=0.082, p<0.01) in the behavioral intention was significant; however, given the small correlation coefficient and the low explanatory power $\left(R^{2}=0.060\right)$, we decided not to incorporate these demographic characters into the further analysis.

### 5.2. Confirmatory Factor Analysis

The measurement model was tested using CFA. Following Anderson and Gerbing (1988) [57], we examined measurement items for the endogenous and exogenous constructs so as not to confound the measurement. The fitness of the model was assessed by five goodness of fit indices, including chi-square statistics, CFI, IFI, GFI, NFI, and RMSEA. The fit statistics and recommended values (Hair, 2009; Hartwick and Barki, 1994) [58,61] are reported in Table 3. The items measuring the independent variables all yielded significant loadings on their respective constructs.

The reliability was assessed by indexes of the factor loading, Cronbach’s α, and composite reliability (CR). According to Hair (2009) [58], outer loading for the indicators above 0.7 was considered good reliability. The internal consistency reliability was measured using Cronbach’s α, composite reliability (CR). Referring to Bagozzi and Yi (1988) [62], the recommended values for both should be above 0.7. The reliability analysis results of this study are listed in Table 4. All factor loading exceeds 0.7 (good reliability), suggesting good internal reality. CR and Cronbach’s α values for all constructs are larger than 0.7, indicating good internal consistency reliability.

The validity of the measurement model is assessed based on the convergent validity and discriminant validity. The convergent validity is measured based on the average variance extracted (AVE). The recommended values for AVE should be ≥0.5 (Fornell and Larcker, 1981) [64]. The discriminant validity is assessed based on the cross-loadings. As suggested by Bagozzi and Yi (1988) [62], the square root of the AVE from the construct should be greater than the correlation shared between the construct and other constructs in the model. The convergent validity and the discriminant validity results of the constructs are listed in Table 5 and Table 3, respectively. Based on the results, criteria for both convergent validity and discriminant validity are met, indicating good model validity.

### 5.3. Structural Model Testing

**Figure 1:**
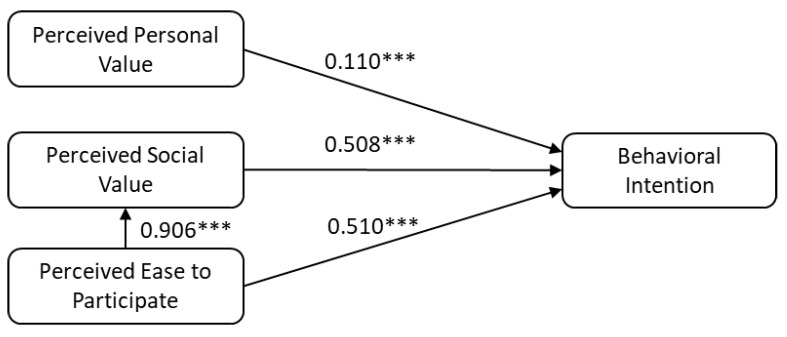
Structural equation modeling analysis results (*** *p* < 0.001, ** *p* < 0.01, * *p* < 0.05).

The structural model is tested to confirm the relationships between the factors as hypotheses. The correlation and the significant level of all relationships are demonstrated in Figure 1. All the hypotheses are supported in the model. Among the antecedents of freshmen college students’ pro-environmental behavioral intention, process ease of use and public values have almost identical large effect sizes on the behavioral intention, with correlations of $\beta_{1}=0.510,\;\;\beta_{2a}=0.508,$ respectively. Furthermore, these two constructs are highly correlated with a correlation coefficient $\beta_{3}=0.906$. Surprisingly, the effect size of personal values on behavioral intention is small to medium $\left(\beta_{2b}=0.110\right)$ according to the guidelines by (Cohen, 1992) [65]. Finally, according to $R^{2}$ square, 82.90% of the college students’ pro-environmental behavioral intention can be explained by the proposed model.

## 6. Discussion

### 6.1. The Impact of Values on College Students’ Pro-Environmental Behavior Intention

The results of the present study confirm the modified TAM as a framework for understanding college students’ motivation in pro-environment activity. Perceived personal values, perceived social values, and perceived ease to participate accounted for a large proportion of the variance in the pro-environmental behavioral intention. There are three findings worth mentioning.

In aligning with previous studies (Belschak and Den Hartog, 2010; Righetti et al., 2015) [25,66], social values are identified as the driving motivation of college students’ behavioral intention in pro-environment activity. Different from the social norm, social values influence behavior intention in a more active way. Thus, it is self-explanatory that, compared to the correlation (0.29) between descriptive norm and intention in the study by De Leeuw et al. (2015) [4], the correlation (0.508) between perceived social values and behavioral intention is larger in the present study. This comparison highlights that to promote pro-environmental behaviors among college students, it is pivotal to shape students’ prosocial values rather than initiating direct intervention. The shaping of prosocial values is more likely to lead to future pro-environmental behaviors.

In addition, the perceived ease of participation is positively related to both the behavioral intention and the perceived social values. Similar to the construct of perceived control in De Leeuw et al. (2015) [4], the correlation between perceived ease of participation and behavioral intention in pro-environmental activity is significant and large. This finding stressed the importance of creating conditions to facilitate environmentally friendly behaviors and remove the participation barriers.

Next, the correlation between perceived personal values and behavioral intention is smaller but cannot be ignored. This finding is congruent with the result of the study by Shafiei and Maleksaeidi (2020) [1] that personal values (e.g., self-efficacy) are significantly related to pro-environmental behavior. Differently, Shafiei and Maleksaeidi (2020) [1] also implied that rewards are negatively correlated to behavioral intention. These contradicting results do not necessarily mean that person’s gains from the pro-environmental activity are limited, but it may be the result that college students are not fully aware of these gains. In practice, pro-environmental activity can bring numerous benefits (e.g., self-efficacy, knowledge, teamwork building), but they might not be clear to college students. In this regard, we highlighted that it is important to better link perceived personal values to behavioral intentions to promote pro-environmental behaviors among college students.

### 6.2. Theoretical Implications

The theoretical contribution of this study is three-fold. We firstly contribute to the pro-environment literature by using a cognitive theory approach to achieve a more nuanced explanation of the underlying motivation of how students’ behavioral intentions towards pro-environmental activities is shaped by their values. The individual-focused behavior intention can be a reference to promote pro-environmental behaviors among the massive public.

Second, this study provides a prototype framework to incorporate values with TAM to probe into the pro-environmental behavior intentions. Compared to other constructs such as competence, we highlighted that behaviors are rooted in values (Bardi and Schwartz, 2003) [23] and exploring the influence of values can provide nuanced insights into the whole cognitive process that shapes individuals’ behavior.

Third, while existing studies posited that prosocial and pro-self are two conflicting kinds of social values orientation (Hilbig et al., 2014; Kaiser and Byrka, 2011) [24,26], we separately investigated their influence on motivating pro-environmental behavior and argued that social values and personal values may not always be conflicting. Pro-environmental activity can promote social values and well-being in addition to increasing personal values (e.g., efficacy, knowledge building, teamwork in the long run). As such, this study also contributes to a better understanding of the concept of values and how they shapes one’s behavior.

### 6.3. Practical Implications

The study has three major practical implications. The first implication comes from the significant relationship and large correlation between the perceived social values and pro-environmental behavioral intention. This result implies that prosocial values are a driving force for college students’ motivation in pro-environmental behaviors. In this regard, for universities and higher-education institutes, it is important to continue shaping students’ prosocial values so as to promote pro-environmental behaviors and a more sustainable environment.

Second, the personal values could exert a positive influence on pro-environmental activity as well. This in turn highlights the need to create personal values from pro-environmental activity. It is often the case that college students are more aware of the cost (e.g., time) of pro-environmental activities, but fail to realize that they may benefit (self-efficacy, academic performance, and careers) from participating in such activities in the long run. Therefore, for instructors in higher education, it is necessary to exploit pro-environmental activity to create meaningful values for college students and make them fully aware of it.

Finally, the perceived ease of participation also has a significant and positive influence on the behavioral intention in pro-environmental activity. This shows that higher education institutions can offer more facilitated conditions, well-described guidance, and more comprehensive pro-environmental action knowledge to promote the perceived ease of participation so as to promote the pro-environmental behavioral intention.

### 6.4. Limitations

This study is not without limitations. First, it is worth noting that the central motivation of this study is to probe into the intention of freshmen students in pro-environmental behaviors so that higher education could develop strategies for education intervention; thus, we used the construct behavioral intention rather than the actual behavior to benchmark. Nevertheless, the cognitive study argues that intention is only weakly correlated with the actual behavior (e.g., 0.21, see De Leeuw et al. (2015) [4]). Thus, future studies are encouraged to explore the theoretical lens of other cognitive theories (e.g., theory of planned behavior, decomposed theory of planned behavior) to further link behavioral intention to the actual behaviors.

Second, the proposed model is empirically tested using self-reported survey data from freshmen students from one university. It is worth mentioning that the results from this study should be interpreted with caution. However, we argued that the research setting in this study is deemed sufficient for the following reasons. First, the university that we recruited respondents from is a diversified university with students from all provinces in China. Thus, we posited that the pro-environmental behavioral intention of students in other universities might be shaped by similar values. Second, since we only recruited freshmen students as respondents, we argued that, compared to other college students, they are less likely to be affected by other influences from the universities (e.g., education orientation, universities’ cultures). Finally, though different universities are prone to exert different influences on student’s value shaping, we argued that these influences are not exerted directly but through the mediating of their values. Thus, with all three arguments above, the model used in this study was deemed validated. Nevertheless, we also recognized the need to recruit respondents from different universities to better reveal universities’ influence on shaping college students’ pro-environment behavior.

The third limitation of the present study resides in not incorporating individual characteristics in the pro-environmental behavioral intention modeling. We realized that in literature, individual factors are widely considered as variables influencing individual behavior (Alonso et al., 2019) [67]. For instance, rural residents may place a higher priority on the environment and may have higher pro-environmental behavior intentions (Huddart-Kennedy et al., 2009) [60]. Gender may also be a predictor of behavioral intention. In particular, women are associated with more intense behavioral intention in pro-environmental activities (Parrado et al., 2013) [59]. Again, we argued that the influence of these predictors is not direct, but through the mediating effect of the values. Still, to further investigate how these individual factors shape an individual’s values, it is necessary to incorporate these individual factors in future modeling.

Finally, the present study uses an individual-focused cognitive approach to explore freshmen college students’ pro-environmental behavior intention; the findings of this study should be interpreted with caution. The actual behavior is prone to alteration by social norms (Czajkowski et al., 2017) [20]. Therefore, future studies are encouraged to incorporate social norms into the proposed model to provide nuanced insights into students’ actual pro-environmental behaviors.

## 7. Conclusions

College students are significant people who need to face and cope with future environmental changes; it is necessary to probe into how their behavior intentions towards pro-environmental activity are shaped. The present study provides a prototype study exploring an important motivation of students’ pro-environmental behavioral intentions through combining the lens of values and the TAM. Specifically, we incorporated perceived social values and perceived personal values with TAM constructs to explore the motivation of freshmen college students’ behavioral intention in pro-environmental activities. In terms of data, we controlled for the education intervention from higher education and chose freshmen students as the targeted respondents because we were interested in exploring the initial values of the students. The proposed research model was validated through a survey of 245 college students at a university in China. Analytical results suggest that among the two conflicting constructs, perceived social values takes the leading role in motivating college students’ pro-environment behavioral intention. In the meantime, the correlation between perceived personal values and behavioral intention is also significant, suggesting that nudging students’ pro-environmental behavior should also take personal interest into consideration. Perceived ease of participation also imposes the main influence on behavioral intention. These results imply that college students in China have strong prosocial values, and higher institutions should continue guiding college students to promote pro-environmental behaviors by continuing to shape their prosocial values and creating personal values from these behaviors.

## Figures and Tables

**Table 1:** Constructs and measurements.

Constructs with Items		Source
**Perceived Social Values**		
**PSV1**	Pro-environmental behavior is beneficial for social well-being.	Adapted from Twizeyimana and Andersson (2019) [56]
**PSV2**	Pro-environmental will create additional values for family, community, and other relationships.	
**PSV3**	Pro-environmental is good for the environmental capability building against risk.	
**PSV4**	Pro-environmental will protect our future generation to have equal rights for the environment.	
**Perceived Personal Values**		
**PPV1**	Devoting time to pro-environmental activity can improve my overall competence	Adapted from Shafiei and Maleksaeidi (2020), Twizeyimana and Andersson (2019) [56]
**PPV2**	Devoting time to pro-environmental activity can increase my self-efficacy	
**PPV3**	Participating in Pro-environmental activity can be beneficial for future career	
**PPV4**	Participating in Pro-environmental activity can obtain new knowledge, contributing to better academic performance	
**Perceived Ease of Participation**		
**PE1**	Participating in Pro-environmental activity is easy.	Adapted from Davis et al. (1989) [51]
**PE2**	It is easy to become proficient in Pro-environmental activity.	
**PE3**	I can follow all the instructions in Pro-environmental activity easily	
**PE4**	Interacting with peers in Pro-environmental activity is easy	
**Behavior Intention**		
**BI1**	I intend to continue participating in Pro-environmental activities in the future.	Adapted from Davis et al. (1989) [51]
**BI2**	I will enjoy participating in Pro-environmental activities in the future.	
**BI2**	I will strongly recommend that others participate in Pro-environmental activity	

**Table 2:** Descriptive statistics for the sample.

Items	N	%	Mean	S.D.
Gender				
Female = 1	143	58.37%		
Male	102	41.63%		
Home Location				
Urban Area = 1	113	46.12%		
Rural Area	132	53.88%		
Academic Disciplines				
Science or Engineering = 1	164	66.94%		
Business or Management	74	33.06%		
Age (age 18)	245		18.72	0.72
Perceived Social Values	245		4.14	1.04
Perceived Personal Values	245		2.88	1.11
Perceived Ease of Participation	245		4.17	1.06
Behavior Intention	245		4.13	1.05

**Table 3:** CFA model estimates.

Model Fit Indices	Results	Recommended Value (Hair, 2009; Hartwick and Barki, 1994) [58,61]
$\mathrm{Chi}-\mathrm{Square}\;\mathrm{statistics}\;\chi^{2}/df$	2.878	≤5
CFI	0.978	≥0.9
IFI	0.978	≥0.9
GFI	0.947	≥0.8
NFI	0.967	≥0.9
RMSEA	0.061	≤0.08

**Table 4:** Reliability and convergent validity analysis.

Constructs	Items	Factor Loading	CR	α	AVE
Perceived Social Values (PSV)	PSV1	0.925	0.956	0.955	0.843
	PSV2	0.897			
	PSV3	0.921			
	PSV4	0.903			
Perceived Personal Values (PSV)	PPV1	0.881	0.952	0.951	0.831
	PPV2	0.939			
	PPV3	0.919			
	PPV4	0.933			
Perceived Ease of participation (PEP)	PEP1	0.884	0.934	0.938	0.779
	PEP2	0.886			
	PEP3	0.841			
	PEP4	0.918			
Behavior intention (BI)	BI1	0.947	0.936	0.946	0.831
	BI2	0.914			
	BI3	0.872			

Note: According to Nunnally (1978) [63], a Cronbach’s α above 0.7 is considered a good reliability.

**Table 5:** Discriminant validity analysis.

Constructs	1	2	3	4
Perceived Social Values (1)	**0.918**			
Perceived Personal Values (2)	−0.854	**0.912**		
Perceived Ease of participation (3)	0.906	−0.812	**0.883**	
Behavior Intention (4)	0.786	−0.786	0.773	**0.912**

Note: Bold figures are the square root of AVEs.

## Data Availability

Data sharing not applicable.
